# Supplementary figures and images for: HIV-1 capsid stability enables inositol phosphate-independent infection of target cells and promotes integration into genes
Source: PLoS Pathog. 2023 Jun 2;19(6):e1011423. doi: 10.1371/journal.ppat.1011423 (PMC10266667; doi:10.1371/journal.ppat.1011423)

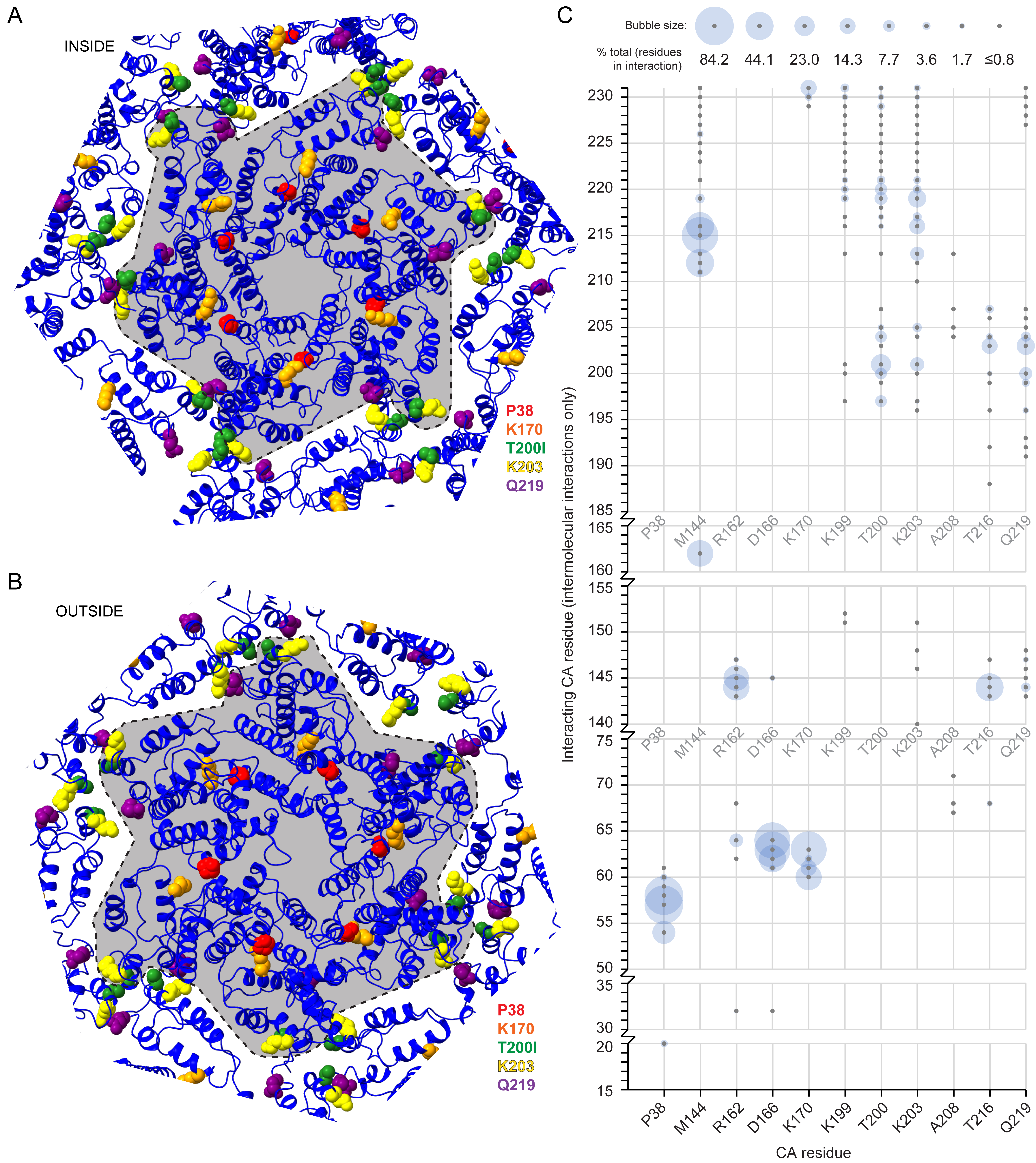

Supplement: S1 Fig — (A and B) Enlarged versions of the right panel of Fig 1A and 1B that portrays the inner (A) and outer (B) surface of an atomic model of the HIV-1 capsid (PDB 3J3Y). (C) Intermolecular CA interactions of IP6-dependent and suppressor residues using an atomic resolution model of the HIV-1 capsid (PDB 3J3Y). The y-axis displays CA residues identified by UCSF Chimera predicted to form intermolecular CA contacts. Bubble size is indicative of frequency of the intermolecular interaction in the model. (TIF) [file ppat.1011423.s001.tif]

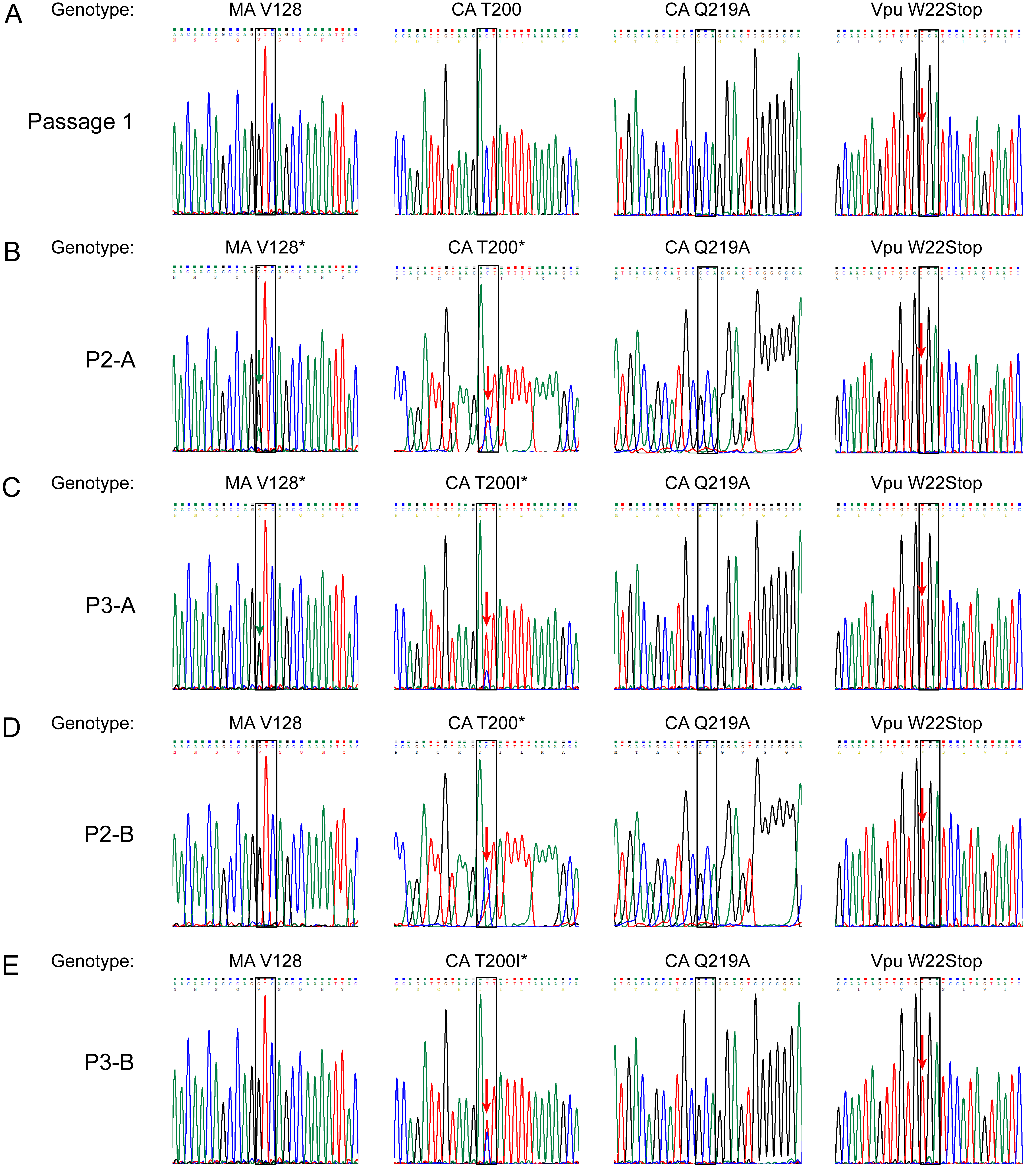

Supplement: S2 Fig — (A—E) Sanger sequencing chromatograms at the indicated HIV-1 loci during passage 1 (P1) and subsequent passages (P2 and P3) of the HIV-1R9 CA Q219A (25 ng p24). P2-A and P2-B are derived from the same P1 HIV-1R9 CA Q219A virus shown in Fig 3A. P3-A and P3-B originate from virus released from P2-A and P2-B, respectively. (TIF) [file ppat.1011423.s002.tif]

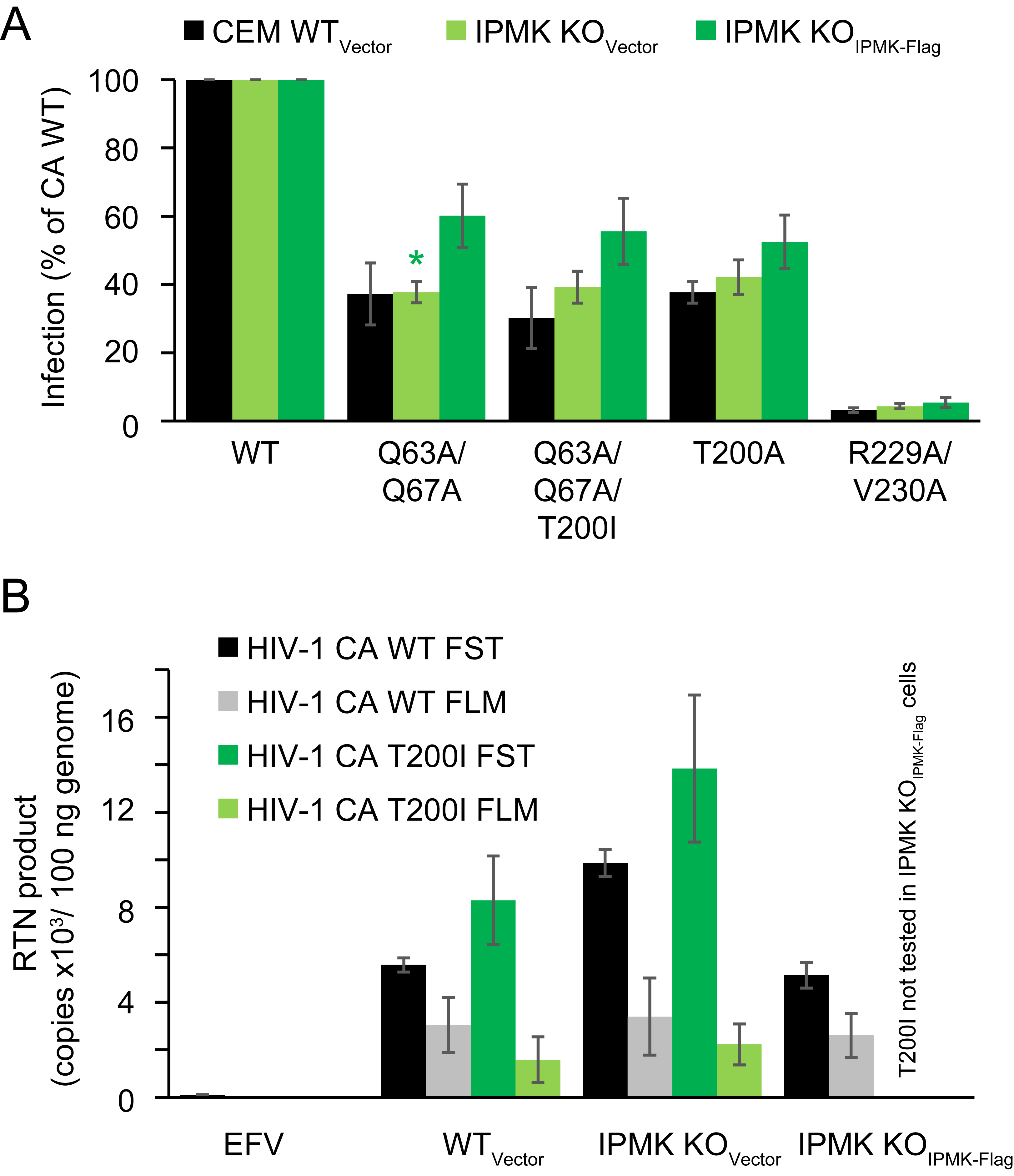

Supplement: S3 Fig — (A) Target cell infection of WTVector, IPMK KOVector, or IPMK KOIPMK-Flag CEM cells by flow cytometry of the indicated HIV-1GFP CA mutants. (B) RTN at 8 h by qPCR in the indicated CEM cell lines. (TIF) [file ppat.1011423.s003.tif]

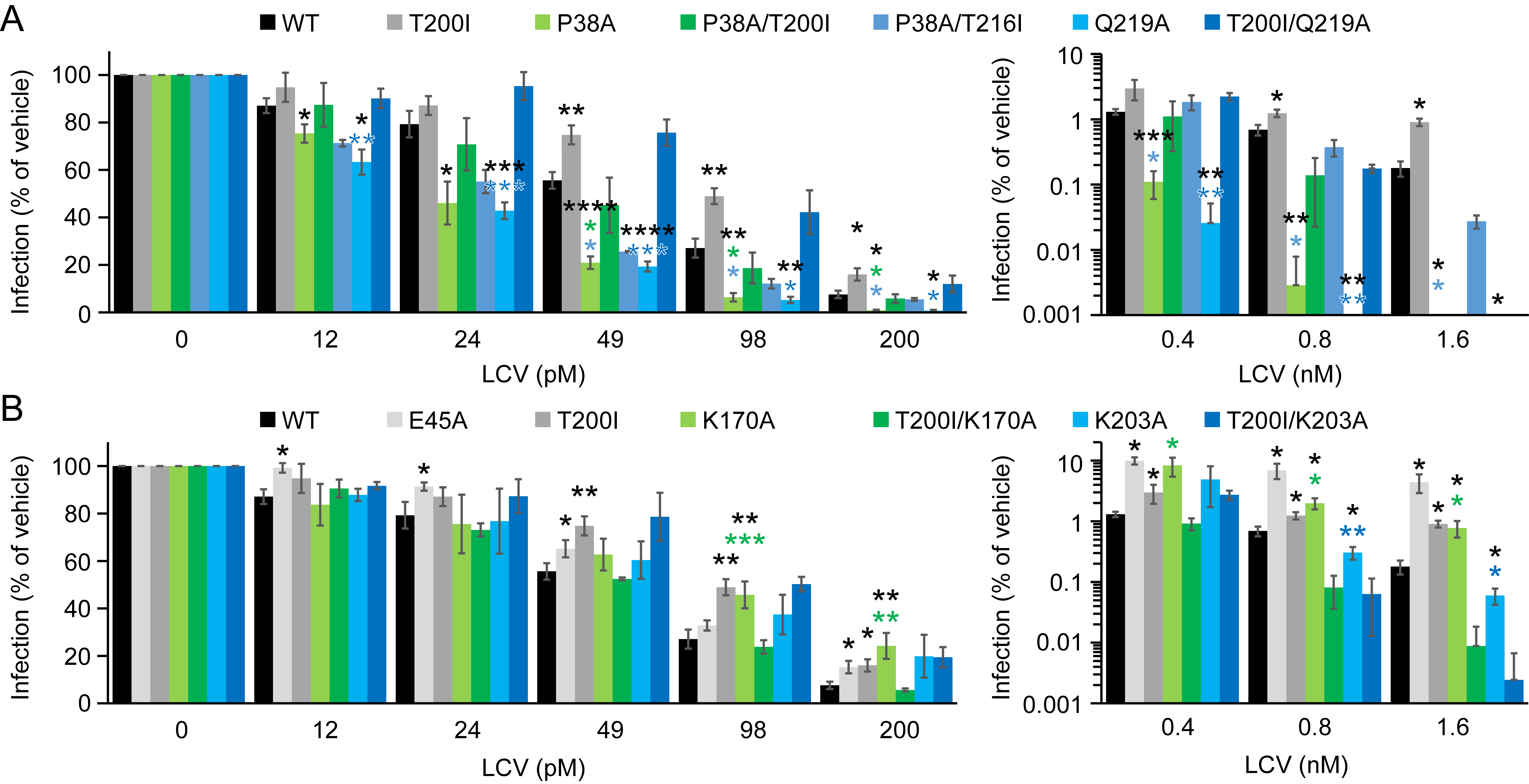

Supplement: S4 Fig — (A) LCV inhibition of the indicated HIV-1GFP CA mutant infection of WT CEM cells. Black asterisk(s) show statistical significance relative to HIV-1GFP WT. Colored asterisk(s) denote significance of the indicated single mutant compared to the respective colored double mutant. Values shown are the average of at least 3 independent experiments. Significance levels: p < 0.05 *, p < 0.01 **, p < 0.001 ***, and p < 0.0001 ****. (TIF) [file ppat.1011423.s004.tif]

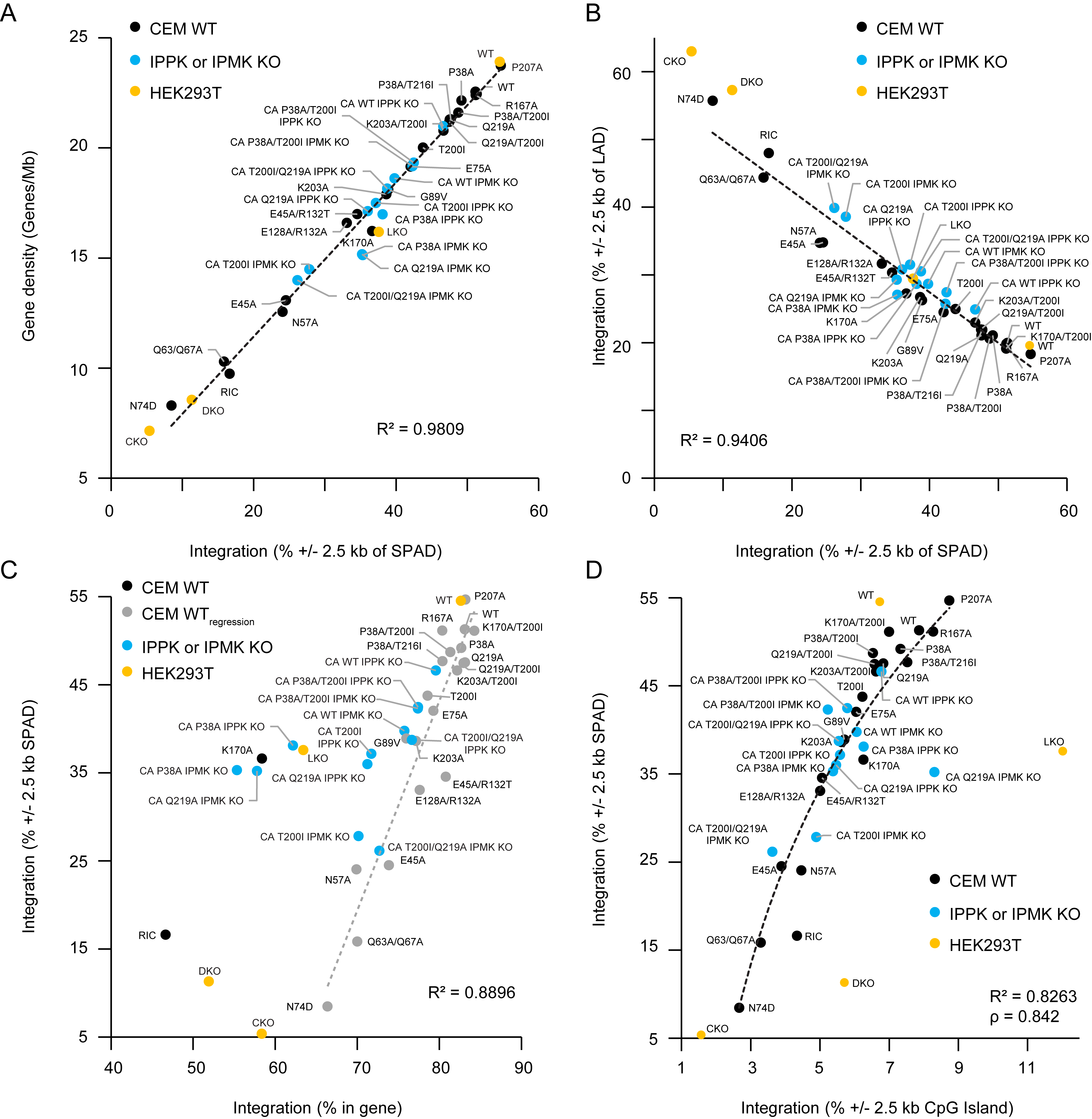

Supplement: S5 Fig — (A-D) HIV-1 ISD results represented by various genomic annotations. Similar to Fig 5 panels E to G, except HEK293T WT and KO cell lines are included for comparison. Abbreviations: LEDGF/p75 KO (LKO), CPSF6 KO (CKO), and LEDGF/p75 and CPSF6 KO (DKO). (TIF) [file ppat.1011423.s005.tif]
