# Supplementary material for: HIV-1 capsid stability enables inositol phosphate-independent infection of target cells and promotes integration into genes
Source: PLoS Pathog. 2023 Jun 2;19(6):e1011423. doi: 10.1371/journal.ppat.1011423 (PMC10266667; doi:10.1371/journal.ppat.1011423)
Supplement: Attachment — Submitted filename: Response_to_Reviewers_FINAL.docx [file ppat.1011423.s018.docx]

**Response to reviewers:**

*We thank the reviewers for their collective insights about the contents of the manuscript. Please refer to the revised manuscript with track changes enabled for line and page numbers used within following rebuttal. All reference numbers correspond to those used in the resubmitted, revised manuscript.*

**Reviewer #1:**

We have made several changes in response to the noted criticisms.

**Major Issues:**

Reviewer #1: The most significant issue is Figure 4A. As it stands based on the colour scheme, the T200I mutant does not form stable cores. It is possible this is an error (as two of the colour schemes in the key do not appear in the graph), but if it is not, this undermines the entire mechanistic argument that T200I provides a stabilisation effect to the capsid.

Regrettably, the legend in Figure 4A contained a significant error; this has been corrected. We appreciate the identification of the mistake.

It would be preferable to see some more compelling evidence regarding lattice stability. Electron micrographs of the mutants to support whether they have any morphological defects would be useful. In vitro CA assembly studies with IP6 and LCV to show that their relative assembly competencies and responsiveness to these compounds would also be helpful.

The virion morphologies of the panel of mutants (by TEM) were previously determined, as were the CA assembly properties of most of the mutants (PMIDs: 14963157, 12692245, 33115869)). This work does not change the conclusions of the manuscript and therefore wasn’t mentioned or cited: Previous studies have characterized the morphological details of P38A, K170A, K203A, and Q219A CA mutants in the context of HIV-1 particles (PMIDs: 12692245 and 33115869). All of the mutants were shown to contain conical cores. It is important to note that a morphological defect (or lack thereof) in a core does not mean that the capsd is more or less stable. For example, Q63A and E75A particles exhibit a reduced percentage of mature cores but are competent for replication (PMID 23497318). Further, K158A makes primarily cones, but the cones are incorrectly formed (PMID 33692109).

Regarding *in vitro* CA assembly: the assembly of many of these mutants was previously characterized (PMID 14963157). The IP5/6-dependent CA mutants (even the most defective virus, K170A) can assemble into tubes. Some mutants, including R18A and K25N, form cone-like structures *in vitro*. While it might be interesting to study the effects of lenacapavir and IP6 on the assembly of CA mutants, these results would be tangential to our conclusions pertaining to HIV-1 use of IP6 in target cells.

Single particle uncoating assays would be ideal (accepting that this is a specialised imaging technique), but some method to quantify relative capsid stabilities is important given that ‘stability’ features so prominently in the title and the proposed model.

The bulk uncoating assay is well established. Results from this assay compare well single virion imaging-based assays like those developed by the Böcking and Melikyan groups (PMID 29877795 and 27322072). For comparison, PF74 destablizes the viral capsid in both assays (PMIDs 20962083 and 29877795) and, IP6 stabilizes the capsid in both assays (PMIDs 34613803 and 29848441).

To complement this assay and include Q219A and T200I/Q219A mutants in the comparison, we have added panels 4C and 4D. Figure 4C tests the effect of IP6 on core-associated CA following virion permeabilization. The results show that both Q219A and T200I/Q219A capsids are less stable than the WT capsid even in the presence of IP6. This uncoating assay failed to reveal stabilization of Q219A mutant capsids by T200I. In new experiments shown in Figure 4D, we quantified endogenous reverse transcription (ERT) in permeabilized virions for, an assay that depends on capsid stabilization (PMID 33033190 and 33262260). The results showed that addition of IP6 promoted ERT in T200I/Q219A but not in Q219A. Collectively, these results suggest that T200I/Q219A is more stable than Q219A in the presence of IP6. Because the optimal ERT signal for T200I/Q219A was less than 10% of ERT in wild type virions, we think the direct uncoating assay lacked the sensitivity to detect capsid stabilization of the T200I/Q219A capsid by IP6.

The Vpu truncation experiments point to a possible role of BST-2 in promoting Q219A replication. This interpretation should be tested by observing the effect of BST-2 overexpression/depletion.

The inclusion of the Vpu mutant A14L/A18L, which specifically affects BST-2 antagonization by Vpu (PMIDs 27531907 and 30717826), addresses this. In replication assays (Figure 7D-F) of WT HIV-1, Q219A, and T200I/Q219A, we observed enhancement of replication by addition of the Vpu point mutant, indicating that loss of BST-2 antagonism by Vpu promotes replication of both Q219A and T200I/Q219A mutants. This was included in the original manuscript, but we have added both citations (PMIDs 27531907 and 30717826) to the revised manuscript to support the conclusion regarding BST-2 antagonism.

Figure 8 needs to be completely remade. A flow of information that starts in the middle, immediately diverges, and mirrors itself with multiple pathways that get further away from each other, but need to be compared, is a poor way to compare and contrast scenarios. The details of superscripts (T200I label reverses the steps?), dashed lines, colours, are too confusing.

To help readers interpret Figure 8, the figure has been divided into 2 panels (A and B) corresponding to WT and IP5/6-depleted cells. Further, the steps affected by T200I are now denoted by a turnstile symbol. The figure legend has been edited to account for these changes and some of the colors have been changed to improve readability.

**Minor Issues:**

Reviewer #1: Line 160 – WT is not presented in the figure

WT is present in the figure and is one of the reference values to calculate the fold change (FC). To clarify this, we have added “The fold change from the y-axis represents the fraction of HIV-1-infected cells for the indicated cell line divided by the percentage of infected cells of the denoted control cell line.” to the figure legend. Further, we have added “fold change” above the legend in panel 2A.

Line 181 – Why was Q219A chosen for spreading infection experiments, and not the other mutations?

The Q219A mutant is less impaired than either the K170A or the K203A mutant. Because our group has previously published a suppressor mutation for P38A (T216I) (PMID 22515365), we chose Q219A because we thought that the probability of recovering a suppressor mutation would be higher with a less severely impaired mutant.

Line 216 – ‘a subset’ of mutants was assayed. Why was P38A omitted from Figure 3D? It is present in all other panels in Figure 3.

In Figure 1D, we showed that P38A reverse transcription in IP5/6-depleted cells is rescued by T216I. To make this clearer to the reader we have added the statement “Addition of T216I to P38A restored RTN in IPMK KO cells to near that in WT cells, demonstrating that the defect is reversible.” to the results. Surprisingly, while T200I enhanced the infectivity of both K170A and K203A, it reduced the infectivity of P38A and had no significant effect on Q219A infectivity (Fig. 3B). Therefore, we contend that analysis of P38A/T200I RTN in target cells would not alter the conclusions regarding suppression of the other mutants. In any case, we emphasize that the most important part of this figure, panel 3C, shows that T200I partially relieves target cell IP5/6 dependence for each mutant, including P38A.

Also, infection is plotted on a log axis, but reverse transcription on a linear axis (Figures 1 and 3). Given that some of the infectivity defects occur over 5 logs, similar defect levels in RT are difficult to discern on a log axis.

With correct gating, our flow cytometric assay for HIV-1 infection has no detectable background. By contrast, the background of reverse transcription in cells ranges from 0.5-1.5% (as determined by titering the virus on cells and performing the qPCR assay). The assay background in the reverse transcriptase assay in cells emanates from residual contaminating plasmid DNA from transfection (the small amount that survives the DNAseI treatment). Thus, the reason for the difference in axis scale is that the target cell infection assay (flow cytometry for GFP) is substantially more sensitive than the RTN assay (comparing an assay that has >5 logs of sensitivity to one that has <3 logs is not appropriate).

Line 274 – ‘…K170A mutant, which contains a highly unstable capsid,…’. This statement is not substantiated by any relative stability measurement.

We have added the requisite citations for this statement in the revised manuscript (lines 318-319).

Line 291-294 – Rewrite this sentence, it is difficult to follow.

This sentence now reads: “Integration by WT and the nonimpaired P207A mutant [15,35] HIV-1 was nonrandom, with >80% and 50% of proviruses occurring in genes and SPADs, respectively (Figure 6A and B, Tables S2-3).”. This makes it clear which annotation is being referred to in the sentence.

Line 315 – ‘Nonetheless, HIV-1 pre-integration complexes (PICs) targeted SPADs at much greater frequency than CA N74D (Figure 6B)’. This sentence is unclear in this context.

We have clarified this sentence to say: “Nonetheless, HIV-1 pre-integration complexes (PICs) of the CA K170A mutant targeted SPADs at a much greater frequency than did the CA N74D mutant (Figure 6B).”

Line 332 – 334 – This sentence required multiple readings, remains unclear and the following conclusion is uncompelling. It is difficult to see how T200I counteracts defects in integration targeting when it increases SPAD integration for P38A and decreases it for Q219A in the IPMK KO cells.

We modified the sentence to read “In each IPPK KO and IPMK KO cell line, addition of CA T200I to HIV-1 CA P38A and CA Q219A restored integration near SPADs at or near the level of CA WT or CA T200I, respectively (Figure 6D, Table S4).”.

For integration in IPPK KO and IPMK KO cells, integration into genes was rescued by addition of T200I to the level of WT HIV-1 for both Q219A and P38A (Figure 6C). Regarding integration near SPADs, we observed T200I rescued P38A integration targeting in IPMK KO cells to the level of WT HIV-1 (Figure 6D). Conversely, the combination of T200I/Q219A rescued integration near SPADs to the level of T200I (Figure 6D). As T200I by itself imparts an integration targeting defect in WT and IP5/6-depleted cells (Figure 6A-D), it is always possible that integration could be rescued to the level of T200I for the mutant or WT. We observed a similar result for target cell infection by all the T200I mutant combinations (Figure 3B). Thus, T200I appears to be imparting a dominant defect on some of the mutants.

Continuation from the point above: And again, why were two of the mutants (K170A and K203A) omitted from this experiment?

K170A and K203A have integration targeting defects in WT cells that were remedied by the T200I suppressor (Figure 5A and B), thus expanding the analysis of these mutants in the KO cell line seemed unnecessary. Moreover, both mutants are so poorly infectious in WT cells and even further impaired in KO cells. Therefore, obtaining sufficient integration events in IP5/6-depleted cells for meaningful analysis would not have been feasible.

**Reviewer #2:**

We appreciate the strong, positive feedback.

**Minor Issues:**

Reviewer #2: 1. Figure 1. The legend is confusing; (F) refers to quantification of early RTN produces but this is labelled (D) in the figure. Likewise, (D) and (E) refer to positions of altered residues mapped onto the hexamer or three-fold lattice but in the figure this is (E) and (F). Perhaps (D) and (F) are mixed up?

This was an unfortunate error; we have corrected the legend.

2. Was the passage (Figure 3A) of Q219A done in WT CEM cells? If so, why was T200I selected given that it does not increase infection by Q219A (Figure 3B)?

In the results section we have clarified that this experiment was done in WT CEM cells (line numbers 189-190). We agree that this is a bit puzzling and commented on it in the original discussion (lines 544-548). From an infectivity standpoint, one might expect both P38A and Q219A to be capable of replicating, as their target cell infectivity defect is quite minor in WT CEM cells. We suspect that the reason for the difference between Q219A and T200I/Q219A is that a spreading infection is more biologically complex (Figure 3A) than the single round target cell assays performed in Figure 3B. It is possible that the limited innate signaling capacity of CEM cells has an impact (restriction factors or metabolic changes). It could also be that the metabolism in the uninfected cells is altered due to co-culturing of uninfected cells with infected cells. It seems possible that IP6 levels may be lower in uninfected cells due to co-culturing with the unhealthy HIV-1-infected cells (which is what we discuss) resulting in a significant loss of infectivity for the Q219A virus. We do not comment much more on this subject as the reasoning is highly speculative and few details exist in the literature to support such hypothesizes.

3. Line 230-231: As the FLM RTN signal is so weak for K203A and K203A/T200I, I think it should also be considered that any difference will be difficult to determine. Therefore it doesn't necessarily mean that T200I must be enhancing a step post-RTN. This data alone isn’t really enough to support the assertion that IP5/IP6 likely affects other stages of target cell infection, as stated in Lines 234-235.

We agree with these comments. The phrasing of line 230-231 in the original manuscript have been softened by adding the word “might”. The sensitivity of the target cell RTN assay is not sufficient to make the claim in lines 234-235 of the original manuscript, and we have removed these lines.

4. Figure 3D. Given that other experiments were done with P38A, it’s a pity data isn’t shown for this mutant here. As P38A is arguably one of the more infectious mutants, its inclusion here would be of interest.

In Figure 1D, we showed that P38A reverse transcription in IP5/6-depleted cells is rescued by T216I. To make this clearer to the reader we have added the statement “Addition of the T216I to P38A restored RTN in IPMK KO cells to near that in WT cells, demonstrating that the defect is reversible.” to the results. Further, T200I makes P38A less infectious. It isn’t clear how this one mutant (P38A) would change the conclusions of the manuscript. In any event, we emphasize that the most important part of this figure is panel 3C, which shows that T200I partially rescues IP5/6-dependence for each mutant (even P38A).

5. Figure 4A. I think the legend is incorrect; presumably the green circle and solid line is T200I? The blue circle solid and dotted lines are presumably Q219A and Q219A/T200I respectively?

We regret this unfortunate error. It has been corrected.

6. Figure 4B. The uncoating experiment was carried out in the absence of IP6 but it would be useful to know whether there is any difference in the presence of IP6, maybe at longer time points?

Since the WT core is extremely stable when IP6 is included in our uncoating assay, we do not include IP6 in the uncoating reaction when testing for a hyperstable HIV-1 core. The uncoating reaction lacking IP6 is extremely sensitive at detecting increased capsid stability as is the case for T200I (example PMIDs: 11991995, 19914170, 22515365, 23097435, 23497318, 34613803). Thus, for what we were testing, the uncoating assay was useful.

In the revised manuscript we have used permeabilized virions to assay uncoating of WT, T200I, Q219A, and T200I/Q219A cores in reactions containing various IP6 concentrations (Fig. 4C). This assay is less sensitive than the uncoating assay using purified cores; nonetheless, we readily observed core stabilization by IP6 using this modified assay for both WT and T200I. This experiment demonstrated that the capsid of T200I/Q219A is less stable than the WT capsid. Further, IP6 stabilizes both WT and T200I capsid apparently to the same extent at the IP6 concentrations tested (IP6 concentrations that are optimal for the WT capsid). Curiously, IP6 failed to detectably stabilize either Q219A or T200I/Q219A cores in the assay.

To complement the direct uncoating assay, we performed endogenous reverse transcription reactions in permeabilized virions. This result showed that T200I cores are poorer at reverse transcription than WT cores, thus explaining the mutant’s reduced infectivity and corroborating its reduced reverse transcription in target cells. Secondly and most importantly, the endogenous reverse transcription assay revealed a difference between Q219A and T200I/Q219A cores. By contrast to Q219A, T200I/Q219A particles exhibited substantial reverse transcription in the presence of capsid-stabilizing concentrations of IP6, suggesting that the double mutant has a more stable capsid than the Q219A single mutant.

7. Lines 303-304. “A spectrum of ISD severities…based on which aspect(s) of CA biology are affected by the mutant”. It is unclear what specific data is being used to draw this conclusion. Please clarify which aspect of CA biology each mutant is affecting.

The aspect(s) of CA biology each mutant affect is cited in the introduction (lines 57-62 and 70-72). CA mutants used for the integration site sequencing and their effect(s) on CA biology are contained within the results as well (lines 332-349). We have added the clarification to lines 303-304 of the original manuscript along with references to previous reports that have observed the same affect(s): “Thus, we observed a spectrum of ISD patterns for the various CA mutants, with those exhibiting impaired nuclear entry showing less pronounced integration targeting changes than mutants with that affect CPSF6 binding [2,6–8]”.

8. Lines 313-316. Please explain the significance of K203A showing a greater %gene defect than N74D but a smaller SPAD defect and what would cause this?

The reviewer is describing K170A which has the described defect relative to N74D. K203A has a lesser defect in integration targeting than N74D or K170A. The K203A defect could result from having a slightly more stable capsid than K170A, but this is conjecture and probably not possible to test. We ascribe the K170A defect (which is a new integration site distribution pattern) to result from an absence of CA on the pre-integration complex (discussion lines 520 to 530). This effect on integration site distribution is recapitulated by P38A and Q219A integration into the genomes of IP5- and/or IP6-depleted cells. Since P38A and Q219A show characteristics of complete capsid instability in IP5/6-depleted cells (failure to reverse transcribe DNA that is rescued by the capsid stabilizing mutation T200I), we suggest in the discussion that the phenotype is due to a lack of CA on the pre-integration complex.

9. Lines 327-332. What is the basis for describing the integration of IP5/6-dependent mutants in IP5/6-depleted cells as substantially affecting %genes but only modestly affecting SPAD? The data in Fig6C&D suggests both are equally significantly affected?

Integration into SPADs and genes is reduced under the conditions that the reviewer describes. This description in regard to integration near SPADs was meant to be relative to WT and N74D, however this distinction does not matter. We have edited the text to reflect this by removing the word “modestly” which now reads: “Conversely, relative to WT virus, integration into SPADs was reduced by IP5 and/or IP6 depletion for each IP5/6-dependent CA mutant.”.

10. Figure 6. A conclusion from these experiments is that “upon IP5/6 depletion, IP5/6-dependent mutants have a unique ISD” but is it not the case that they have a similar ISD to unstable mutants? For instance, K170A shows a similar reduction in % gene integration as P38A in IPMK KO cells.

The reviewer is absolutely correct; this was an oversight. The point we wished to make is that the IP5/6-dependent viruses in IP5/6-depleted cells and the K170A mutant have unique integration site distributions. We state this in the discussion, but the point should be made here as well. The sentence now reads “Thus, we conclude that, upon IP5/6-depletion, IP5/6-dependent HIV-1 mutants have a unique ISD akin to K170A integration caused by a complete destabilization of the capsid.”.

11. Related to the previous point, it would be important to test whether K170A shows any further reduction in % gene integration in IPMK KO cells, particularly as this mutant is associated with a reduction in infectivity (Figure 2B).

We agree this would be theoretically nice to test. However, this experiment is not feasible. The loss of infection resulting from K170A by itself made integration site sequencing challenging owing to the low number of integrations. The further reduction in K170A infection in IPMK KO cells is too great to yield a sufficient number of integrations to obtain statistically meaningful data. This is also true for K203A. K203A and K170A are simply not infectious enough in the knockout cells to obtain meaningful data in the integration site distribution assay.

12. An unstable capsid in IP5/6-depleted cells would logically be expected to show defects downstream of RTN because it may collapse after RTN but before integration. Therefore in order to conclude that there are specific integration site defects, rather than merely a linear correlation between stability and the number of proviruses, the proviral DNA levels for mutants in WT, IPPK KO and IPMK KO conditions should be shown (and similar linear correlations tested as in Figure 6E-G).

At no point in the manuscript do we correlate the number of integrated proviruses with reverse transcription, nor do we say there is an integration defect. By contrast, there is an integration targeting defect; integration targeting analyzes where the virus integrates and not the efficiency of integration per se. Given the dearth of reverse transcription for the IP5/6-dependent mutants in IP5/6-depleted cells, it is not clear how this would provide meaningful results (i.e. neither assay suggested by the reviewer is sufficiently sensitive or precise to determine whether there is less integration than reverse transcription).

The experiment shown in Figure 6 was aimed at probing the effects of capsid stability and IP5/6 on steps occurring after reverse transcription. While it is well established that a sufficiently stable capsid is required for reverse transcription, the experiment analyzed only viral events that completed reverse transcription. Therefore, the conclusion that capsid stability influences integration targeting is reasonable and appropriate.

13. In general the section dealing with Figure 6 could be improved by explaining why the phenotypes of certain mutants suggest they impact different aspects of CA biology. I assume the purpose here is to differentiate mutants that impact nuclear import vs integration but it is not clearly spelled out.

The aspect(s) of CA biology each mutant affect is cited in the introduction (lines 57-62 and 70-72) and results as well (lines 341-349). We have added the clarification to lines 350-353 along with references to previous reports that have observed the same affect: “Thus, we observed a spectrum of ISD patterns for the various CA mutants, with those exhibiting impaired nuclear entry showing less pronounced integration targeting changes than mutants with that affect CPSF6 binding [2,6–8]”.

The purpose of the first paragraph pertaining to Figure 6A and B (lines 330 to 353) is to compare the hyperstable capsid mutants, including T200I, to other CA mutants with known integration targeting defects. The section is intentionally brief because the purpose of cataloging the mutants is for comparative purposes. The main point that we want the reader to take home from this section (which the reviewer appreciated) is that K170A along with P38A/ Q219A integration into IP5/6-depleted cells have a novel integration pattern. We can make this claim because we tested so many mutants in our T cell line.

14. Figure 7. A further CA mutation, G208R, was obtained upon passage of T200I/K203A virus. In order to determine if this mutation has actually impacted T200I/K203A fitness it would be important to test whether it alters infectivity of T200I/K203A in WT and IP5/6-depleted cells.

G208R was reported to suppress another capsid mutant H62A as was stated and cited in the manuscript (PMID 21367891). We did not claim that the mutation improved T200I/K203A fitness. However, the acquisition of a mutation during HIV-1 passaging, especially in CA, implies that the mutation is beneficial to the virus. T200I/K203A partially rescues the IP5/6-dependence of K203A. Therefore, determining whether G208R further enhanced fitness of T200I/K203A would not alter the conclusions of our study, so this experiment is currently beyond the scope of the present study.

**Reviewer #3**

We have addressed all the reviewer’s comments.

**Major issues:**

1. The results related to relative stability HIV cores are presented in Fig 4. The graphs in Fig 4A appear to be mislabeled as they do not correspond to the description of these results (see lines 246-249). Based on the description, the solid green line would be T200I rather than Q219A. Also, I cannot tell which mutants are shown by dashed and solid blue lines, but the description indicates that the cores with Q219A and Q219A/T200I mutations could not be purified. Even after the authors correct their careless mistakes, how can one conclude from these results that Q219A/T200I is more stable than Q219A?

Regrettably, we had made an error in the legend. We have corrected it.

T200I/Q219A reverses multiple infection phenotypes imposed by Q219A, including IP5/6-dependence (Figure 3), LCV hypersensitivity (Figure 5), pre-integration complex functionality upon IP5/6-depletion (Figure 6), and importantly HIV-1 replication (Figure 7) all of which argue that T200I/Q219A has increased capsid functionality relative to Q219A. We have added more panels to Figure 4 that help address T200I/Q219A relative to Q219A capsid function *in vitro* (discussed in below in 2.).

2. To somehow justify their claim about potentially stabilizing effects of the T200I change, they compare WT vs T200I CA cores in Fig 4B. These assays compare WT with T200I CA rather than Q219A vs Q219A/T200I. Furthermore, I doubt that this assay is quantitative enough or the observed differences between WT vs T200I CA cores are substantial enough to justify their claim. Did they do control experiments in the presence of IP6 to show that both WT and T200I cores remain similarly stable during 2 hrs? I would suggest using more sophisticated assays (for example see PMID: 27322072) to quantitate effects of the T200I CA change. Once again, what matters is in the difference between Q219A vs Q219A/T200I.

We characterized capsid stability in Figure 4B using an uncoating assay that has been used extensively in the past (non-exhaustive PMIDs: 11991995, 19914170, 19914170, 20962083, 22515365, 23097435, 23497318, 33262260, 34613803). Results using this assay show concordance with newer single virion imaging-based assays developed by the Böcking and Melikyan groups (PMID 29877795 and 27322072) [For example: PF74 destabilizes the HIV-1 capsid in the assays (PMIDs 20962083, 29649444, and 298777950). Further, IP6 (PMIDs 34613803 and 29848441) and CYPA (PMIDs 23097435 and 29877795) stabilize the capsid in the assays.]. Uncoating in the absence of IP6 is appropriate for testing for hyperstable mutants like T200I; this is again standard protocol for testing for a hyperstable capsid and has been used in several published studies (e.g., PMID 11991995, 22515365, 23497318, 19914170).

To complement the uncoating results in Figure 4B (which tests for capsid hyperstability), we have added two panels to Figure 4 (C and D). Figure 4C shows the effect of IP6 on levels of core-associated CA in detergent-permeabilized virions. The results confirm that WT and T200I cores are stable in the presence of 10 µM IP6 or higher. Panel 4C also shows that the capsid of T200I/Q219A is less stable than the WT capsid and that neither Q219A nor T200I/Q219A was detectably stabilized by IP6. Figure 4D uses permeabilized virions for endogenous reverse transcription (ERT), an assay that is more sensitive than that used in 4C. ERT is an excellent assay to address capsid stability of CA mutants as the assay depends on capsid stabilizing concentrations of IP6 (PMIDs 33033190 and 33262260). Results from the ERT assay suggest that the T200I/Q219A capsid is apparently more stable than that in Q219A virions but not as stable than the WT capsid. Further, the T200I particles were attenuated in ERT by ~3-fold corroborating the observed reduction in infectivity and reverse transcription in cells. Collectively, we suggest that these results indicate that IP6 stabilizes the T200I/Q219A sufficiently to promote ERT, but that capsid stabilization is insufficient to detect in our direct assays that quantify CA association with HIV-1 cores.

3. The authors talk exclusively about effects of these mutations on core stability. Did they measure how these substitutions affect virion maturation? I would like to see TEM images of Q219A vs Q219A/T200I as well as WT vs T200I virions. The quantitative comparison of these virions may shed light whether the examined substitutions affect late steps of HIV-1 replication/formation of properly mature, conical cores.

Our paper and its conclusions relate to target cell effects, and we think that bringing in maturation would likely obscure the message. Importantly, TEM of Q219A compared to WT has been previously described in several reports (PMIDs 12692245 and 33115869). The fraction of mature cores in Q219A virions is similar to that in WT HIV-1 particles. Thus, performing this work for T200I and T200I/Q219A seems unnecessary. Perhaps the experiment proposed by the reviewer would be appropriate to test in another study on maturation, however Q219A might not be an appropriate mutant to use for such as study.

4. Their results with lenacapavir are overinterpreted by solely looking at them through a prism of the capsid stability. Did they check how these CA substitutions affect lenacapavir binding to CA hexamers or monomers? These amino acids are not positioned in the inhibitor binding pocket but indirect effects of various substitutions on the inhibitor binding must be given an important consideration. I am not asking to do these additional biochemical experiments, but the authors should markedly tone done their speculation and acknowledge other possibilities. For example, it is well documented that lenacapavir binds pre-formed, stabilized hexamers with substantially higher affinity than CA monomers (PMID: PMID: 32612233). These findings are not considered in the discussion.

We have added the statement “We cannot exclude the possibility that some of the CA mutations affect LCV binding. For mutation affecting the 3-fold interface (T200I, K203A, and Q219A) an indirect effect of the mutation on LCV seems unlikely, since LCV binds at the NTD-CTD interface. While allosteric effects of the threefold interface substitutions are possible, such effects have not been observed on the binding of this family of CA inhibitors, which includes PF74. By contrast, resistance to small molecule capsid inhibitors can occur without affecting their binding. For example, the E45A substitution in CA renders HIV-1 highly resistant to PF74, yet E45A does not appear to inhibit binding of the compound to the viral capsid [30]. ” to the discussion, suggesting that this conclusion is unlikely for CA mutants that affect the 3-fold interface (yet still altered LCV sensitivity).

**Minor Issues:**

Reviewer #3: They state that lenacapavir is in the phase III clinical trial. The drug has already been approved by FDA for clinical use.

We appreciate the reviewer bringing this to our attention. The statement in the text has been updated.

**Reviewer #4**

The reviewer’s suggestions have been incorporated into the manuscript.

**Major issues:**

1) The description of the data and the conclusions are reasonable. However, the figure presentation is difficult to interpret. A solution would be to provide a key of the mutations mapped onto the structure or a depiction of the lattice. The authors attempt this in one figure, but it is too small, and not nearly informative enough. The addition of such mutation maps could drastically improve the readers understanding of the interpretation of the results, and the rational for certain experiments.

We have retained the structural map in image 1 to show where all the mutations tested reside. Using an all atoms HIV-1 capsid model (PDB 6J3Y), we have added new images that highlight P38, K170, T200, K203, and Q219 residues as spheres. These new images were added to Figure 2 as a small image and Figure S1 as a larger version next to the interaction map for selected residues of the capsid. We think that Figures 2A-B and S1A-B allow the reader to identify the location of the residues more clearly in the capsid lattice.

2) The manuscript correctly reports that IP5/IP6 depletion in cells that are being infected does not significantly alter the outcome. If this is true, what is the purpose of enriching IP5/IP6 in the immature virus particle?

Two papers from Leo James lab and one from ours have demonstrated that the purpose of enriching IP5/6 in virions is to facilitate morphogenesis and maturation of Gag so that the mature capsid can assemble (PMIDs 33692109, 36624347, and 33476323). We do not discuss these publications (but cite them) in the paper as understanding IP6 packaging into the virion is unnecessary to understand our study, which is focused on target cell IP6 and its effects on infection.

If IP5/IP6 is needed to purify capsid in vitro, why is it not needed in receiving cells?

In our experience IP5/6 is not needed to purify HIV-1 cores *in vitro* (PMID 11991995 and others). Moreover, the *in vitro* IP6 capsid stabilizing data provide only a part of the story pertaining to capsid stability. These assays are limited to what is included in the assay, and factors in addition to IP6 have been shown to stabilize the capsid *in vitro.* These include host proteins the interact with the 3-fold interface, Sec24C (PMID 33649557), and CYPA (PMIDs 23097435 and 29877795). We discuss this topic in lines 478 to 485 of the discussion of the revised manuscript and state that CYPA and Sec24C can stabilize the capsid. Thus, it seems plausible that the binding of host proteins in target cells and/or the intrinsic stability of the HIV-1 capsid reduce the requirement for target cell IP6 during infection by wild type HIV-1.

Given that the KO's used do not eliminate both IP5 and IP6, is it possible that the remaining levels are sufficient for capsid? If so, how can WT be not dependent, but the mutants used be dependent on IP6.
A simple solution would be to soften some of the language used throughout the manuscript related to this.

An effect of IP5/6 depletion on infection by WT HIV-1 is observed, but it is modest (PMIDs 33476323 and 34613803). Further, an excellent paper by the James lab (PMID 36624347) demonstrates that even marked depletion of IP5/6 (by > 2 logs which puts the cellular concentration of IP6 at <0.4 µM, a concentration that does not stabilize the capsid (PMIDs 29848441 and 34613803)) has no effect on HIV-1 target cell infection suggesting that the metabolites are not essential for target cell infection. By contrast, we show in this study that the effect is quite strong on infection by a subset of CA mutants with unstable capsids. Because IP6 is known to stabilize the capsid *in vitro*, perturbations that destabilize the capsid in target cells, either pharmacologic (PMID 34613803) or genetic (this paper), can increase the dependence on IP6. Further, as we note in the discussion (lines 480 to 486), other cellular factors are able to stabilize the capsid *in vitro* and in cells. All of this together likely explains why the metastable WT HIV-1 capsid does not require IP6, but the unstable mutant capsids require IP6 for target cell infection.

3) Both mutations R18A and K25A are known to prevent or alter assembly of capsid, and subsequent infectivity. How do the stabilizing mutations identified at the three-fold interface respond in the context of R18A? What are the implications of these two mutations in the current study?

We set out to identify capsid determinants that cause HIV-1 to be dependent on target cell IP5/6 that are distinct from the IP6 binding site. K25N and R18A do not test this. Testing whether T200I influences K25N or R18A infectivity and stability might be of interest for another study. K25N and R18A are distinct from the mutants that we identified. Both mutations alter CA assembly *in vitro*, resulting in formation of cone-like rather than tubular structures (PMIDs 14963157 and 33332391), suggesting a qualitative effect on capsid assembly. It is possible that the R18A and K25N capsids may be assembling like K158A (a mutant that disrupts IP6 incorporation into the virion) in virions wherein the capsid appears morphologically like a cone, but the cone is assembled incorrectly (PMID 33692109). Considering these differences, more work on R18A and K25N should be done, but this is tangential to the goal of our current study.

**Minor Issues:**

The authors have not cited multiple manuscripts that detail important aspects of IP5/IP6 interactions with capsid, and the effect of IP5/IP6 cellular depletion on infectivity that are highly relevant to the current study. Please correct this oversight.

We have added PMID 30069050 which pertains to release and assembly and not target cell IP6. However, other manuscripts that deal with IP5/6-depletion during target cell infection have been appropriately cited in the original publication (PMIDs 33476323, 31851928, 32776974, and 36624347).

Lenacapavir is now EU and FDA approved, and goes by multiple names. All names should be indicated early, and then whatever the authors chose to call the compound beyond that is fine.

We thank the reviewer for bringing this to our attention. We have made this updated the manuscript accordingly change (line numbers 36 and 89).

In the discussion of Lenacapavir (last paragraph of the manuscript), Fig. 4 is referenced instead of Fig. 5.

This has been corrected.

Lastly, the size of panels in Fig. 6E-F can be increased to increase legibility. Please double check all figure labeling and in text reference to figures.

We agree that it would be useful to increase the size of Figure 6E-G, but this is not possible without making the figure become unnecessarily cluttered or making the figure fail to fit the journal’s size requirements. Thus, to make the figure easier to visualize, we have increased the size of Figure S5 which contains the same information but includes integration site data from HEK293T WT, CPSF6 KO, LEDGF/p75 KO, and CPSF6/LEDGF double KO cells. All of the plotted data in Figure 6 is included as a Tables S3 and S4. We have checked all figure labeling and text references to figures and the mistakes have been remedied.

6. What was the rational for using Q219A for selections as opposed to the other mutants?

Our group has previously published a suppressor mutation for P38A (T216I) (PMID 22515365). We chose Q219A because we expected this mutant, which has a less severe infectivity impairment than K170A and K203A, would have a greater probability of acquiring a suppressor mutation.

What are the implications of the mutants in this study if they are mapped onto the Pentamer or the Pentamer-Hexamer 3-fold?
This is an interesting question, as the effects of CA mutations on hexamer-pentamer interactions is an unexplored area. A pentamer model (PDB 7URN) recently published by the Pornillo’s lab (PMID 36759579) is shown below. The model uses PDB 4XFX initial model for fitting. The model does not affect our conclusions because the purpose of the residue contacts remains the same (e.g. P38 and K170 form intra-hexamer interactions. K203 and T200 form inter-hexamer interactions. Q219A is at the intra- and inter-hexamer interface). Further, we have not included this image as the initial structure (PDB 4XFX) used to fit the cryo-EM data does not capture relevant known contact information at the 3-fold interface (making the 3-fold interfaces in the model less relevant).

Legend:

Model of HIV-1 pentamer (teal) surrounded by hexamers (blue) from PDB 7URN, PMID 36759579.
P38 in red. K170 in orange. T200 in green. K203 in yellow. Q219 in purple. IP6 is in the central pore of the pentamer.

Typos, poor grammar, or difficult to understand:
Lines: 58

To clarify this line, we have included a sentence after this sentence that describes LADs as: “LADs are genomic regions that bind to the nuclear lamina and are transcriptionally repressed [9].”

291-294

This sentence now reads: “Integration by WT and the nonimpaired P207A mutant [15,35] HIV-1 was nonrandom, with >80% and 50% of proviruses occurring in genes and SPADs, respectively (Figure 6A and B, Tables S2-3).”. This makes it clear which annotation is being referred to in the sentence.

348

This sentence now reads: “However, HIV-1 CA P38A and CA Q219A integration into IP5- and/or IP6-depleted cells (and HIV-1 K170A integration) did not follow this correlation for these two annotations, restricting proviruses to ~35% integrations near SPADs and lowering integration into genes near or below the level of CPSF6 binding-defective CA N74D mutant (Figure 6G and Table S4).”.

462

We have deleted the word “leads”.

424--...IP6 may readily dissociate

We agree and have added the words “may readily” to the sentence.

438--residues published, residues reported

This sentence now reads: “Conversely, T200 forms contacts with I201 and A204, residues reported to stabilize the three-fold interface [41,42].”.
